# The risks and benefits of using social media to engage consumers in service design and quality improvement in Australian public hospitals: findings from an interview study of key stakeholders

**DOI:** 10.1186/s12913-021-06927-x

**Published:** 2021-08-26

**Authors:** Louisa Walsh, Nerida Hyett, Jayne Howley, Nicole Juniper, Chi Li, Belinda MacLeod-Smith, Sophie Rodier, Sophie J. Hill

**Affiliations:** 1 La Trobe University, Melbourne, Australia; 2 Woodend, Australia; 3 Orygen, Parkville, Australia; 4 Albury Wodonga Health, Albury, Australia; 5 Safer Care Victoria, Melbourne, Australia; 6 Northern Health, Epping, Australia

## Abstract

**Background:**

Engaging consumers - patients, families, carers and community members who are current or potential service users - in the planning, design, delivery, and improvement of health services is a requirement of public hospital accreditation in Australia. There is evidence of social media being used for consumer engagement in hospitals internationally, but in Australia this use is uncommon and stakeholders’ experiences have not been investigated. The aim of the study was to explore the experiences and beliefs of key Australian public hospital stakeholders around using social media as a consumer engagement tool. This article focuses on the study findings relating to methods, risks, and benefits of social media use.

**Methods:**

Semi-structured interviews were conducted with Australian public hospital stakeholders in consumer representative, consumer engagement/patient experience, communications or quality improvement roles. Qualitative data were analysed using a deductive content analysis method. An advisory committee of consumer and service provider stakeholders provided input into the design and conduct of this study.

**Results:**

Twenty-six Australian public hospital service providers and consumers were interviewed. Participants described social media being used to: recruit consumers for service design and quality improvement activities; as an online space to conduct consultations or co-design; and, to gather feedback and patient experience data. The risks and benefits discussed by interview participants were grouped into five themes: 1) *overcoming barriers to engagement*, 2) *consumer-initiated engagement; 3) breadth* vs *depth of engagement,* 4) *organisational transparency* vs *control* and 5) *users causing harm.*

**Conclusions:**

Social media can be used to facilitate consumer engagement in hospital service design and quality improvement. However, social media alone is unlikely to solve broader issues commonly experienced within health consumer engagement activities, such as tokenistic engagement methods, and lack of clear processes for integrating consumer and patient feedback into quality improvement activities.

## Background

Social media are online applications and websites that allow users, not just site owners or managers, to create their own profiles, generate and curate their own content, and develop social networks by connecting with other users [1]. Commercially available platforms such as Facebook, Twitter, YouTube, WhatsApp and Slack, and privately developed platforms with functions such as discussion forums or chat, can all be considered forms of social media. Social media is used by health organisations, providers and consumers for a range of functions, including finding information [2, 3], gathering data [3], peer support [4, 5], and creating more equal relationships between providers and consumers [5].

Social media is used to engage a range of health stakeholders in service design and quality improvement (QI) activities. A recent scoping review of 40 studies conducted by members of this author team [6] found that social media has been used in a wide variety of ways by health organisations, service providers, consumers and the general public to engage in health service design or QI activities, or to try and influence change in health services. Social media is used as a place to gather QI-relevant information, conduct consultative activities, collectivise and advocate for change, create networks between people working on projects, and as a virtual setting for collaborative discussions and project work [6]. Despite social media being a suitable place to conduct these activities, the use of social media for stakeholder engagement remains relatively uncommon in hospitals and health services [7, 8].

Existing research into the use of social media to engage stakeholders (including consumers, service providers, policy makers and the general public) in health service design and QI has found a range of risks and benefits. Commonly identified benefits of using social media for stakeholder engagement are improving the efficiency of organisational communication [9–12] and helping participants build good working relationships in health service design or QI activities [13–15]. The risks of using social media for stakeholder engagement include engagement activities being less effective than traditional forms of engagement in service design or QI [10, 13, 14, 16] and the potential for social media communications to cause harm due to issues with privacy, professional behaviour or malicious messages or actions [9–11, 15].

In Australia, involving health consumers (patients, families, carers and communities who are current or potential users of health services [17]) in the planning, design, delivery, measurement and evaluation and improvement of health services is a requirement of public hospital accreditation [18]. There is interest in the use of social media for consumer engagement activities in Australian public hospitals and health services [18–20] but health service representatives have indicated that they need further guidance before undertaking such activities [20]. Additionally, none of the included studies in our recent scoping review were from Australia [6], indicating a gap in knowledge around the experiences of Australian hospital stakeholders around the use of social media as a consumer engagement tool.

The aim of the interview study described in this article was to explore the experiences and beliefs of Australian public hospital consumer representatives and service providers involved in service design and QI activities around using social media as a consumer engagement tool. This article presents the findings from the study which focus on the methods, risks and benefits of social media use.

## Method

Semi-structured interviews [21] were conducted within a qualitative description study design [22] to explore the experiences and beliefs of stakeholders towards the use of social media as a tool for consumer engagement in public hospital service design and QI activities.

### Involvement of stakeholders in the research

An advisory committee of key stakeholders, including healthcare consumers and service providers, provided oversight of the research project, and were involved in the design of the interview guide, the data analysis, and preparing the manuscript. As a result of these contributions, five members of the advisory committee are co-authors on this manuscript (JH, NJ, BM, CL, SR). Members of the advisory committee came from a range of backgrounds. Members had professional and/or lived experience as carers or patients in palliative care, mental health, ICU and critical care, transplantation, chronic disease, consumer engagement and representation, and health communications. Members also had experience delivering or accessing services in a range of public hospital settings, including specialist youth health services, regional and rural settings, metropolitan settings, and through public hospital outreach to the community. The INVOLVE principles were used to guide the involvement of stakeholders in the research [23].

### Recruitment and sample

A convenience sampling method was used [24]. Participants were recruited through the networks and communication channels of the researchers and advisory committee, sharing information about the study through social media (Twitter and Facebook), and through contacting Australian public hospitals, public health networks and relevant health condition and consumer peak bodies to share the study information throughout their own networks and communication channels. People who were interested in participating contacted LW directly and were screened for eligibility. Eligibility criteria were: aged > 18; living in Australia; experience in a consumer representative, QI, consumer engagement/patient experience or communications role in an Australian public hospital; with interest in, or experience of, the use of social media (for any purpose); able to participate in a 60 min interview (face-to-face, telephone or videoconference, depending on the participant’s location and preference).

In this study, consumer representatives were defined as “health consumers who had a specific role within a public hospital to provide advice on behalf of consumers, with the overall aim of improving healthcare ( [17] pg 38)”. Service providers were defined as hospital employees involved in either clinical or non-clinical hospital roles which included responsibilities around conducting service design and QI activities, facilitating consumer engagement in service design and QI, or managing hospital social media communications.

People who were eligible for the study were provided with detailed information and were enrolled once they had completed a written informed consent form. Each participant’s consent was reconfirmed verbally at the start of their interview.

### Data collection

An interview guide was developed to explore the experience of, and beliefs about, social media as a tool for consumer engagement in Australian public hospital service design and QI. The guide was developed in consultation with the advisory committee who also participated in test interviews. Demographic data of participants were also collected – age; gender; hospital role; state located; and name and location of hospital.

LW conducted all interviews with participants. Interviews were audio recorded and transcribed verbatim by LW. During transcription any identifying information about the participant or the hospital(s) they performed their role in was redacted from the transcript. Reflective memos [25] made by LW after each interview were transcribed and used for reflexivity and an audit trail.

### Data analysis

Demographic information of participants was presented using descriptive statistics. Information about hospitals provided by participants (size, service types, location, social media platforms used) was compared with information publicly available on hospital websites.

Qualitative deductive content analysis [26] was conducted, and data was stored and managed, on NVivo 12 [27]. An analysis framework was developed from a priori categories identified through the scoping review [6]. Codes were refined and added throughout the analysis to reflect new concepts that were not identified through the scoping review.

The data analysis was primarily completed by LW, however co-authors contributed to the analysis at early stages of the coding process. At the beginning of the first round of coding, NH tandem coded two interview transcripts alongside LW. This served to both to pilot the framework and also provided an opportunity for a discussion about LW’s initial analysis process. Towards the end of the first round of coding, all authors participated in an in-depth group discussion of one of the interview transcripts to identify views and insights about the data that LW may have missed. These discussions with co-authors were key to reflexivity throughout the data analysis because they gave opportunities for LW to understand alternative viewpoints about findings in the data and the analysis process, which enhanced her ability to appraise and critique her role in the research process as she analysed the data [28]. From a practical point of view, the initial discussions with NH about the use of the coding framework resulted in a shift in LW’s initial coding approach from being highly detailed and specific, to approaching coding more broadly in the first round, and refining the analysis down to more detailed codes and themes in subsequent rounds of coding. The group discussion about a single interview transcript resulted in new themes and codes being added to the analysis framework.

### Ethics approval

Ethics approval for this study was given by the La Trobe University Human Research Ethics Committee, Application ID HEC19427. This study and all methods were carried out in accordance with the Declaration of Helsinki, and the National Health and Medical Research Council’s Australian Code for the Responsible Conduct of Research and National Statement on Ethical Conduct in Human Research.

## Results

Twenty-six semi-structured interviews were conducted between October 2019 and April 2020. Key features of participants are detailed in Table 1.

**Table 1:** Key features of participants

Key features			***n***
Gender	Male		8
	Female		18
Age group	18–25		2
	26–35		3
	36–45		5
	46–55		9
	56–65		4
	66–75		3
Participant role	Consumer representative (CR)		12
	Service provider	Total	14
		Consumer engagement (CE)	5
		Communications (CO)	5
		Quality improvement (QI)	4
State located	Victoria		15
	Queensland		8
	Western Australia		2
	South Australia		1

Participants had experience with a range of social media platforms, the most common platforms used were Facebook (*n* = 23), Instagram (*n* = 14), LinkedIn (*n* = 13) and Twitter (*n* = 11).

Participants came from 18 different Australian public hospital settings. Settings varied from large tertiary and quaternary health services with over 1000 inpatient beds across multiple campuses, to highly specialised hospitals with fewer than 50 inpatient beds. According to Australian Bureau of Statistics classifications [29], 14 of the represented settings were in major Australian cities, three were inner regional, and one was outer regional. There were no remote or very remote hospitals represented in this study.

### Participants’ experiences of using social media as a consumer engagement tool

Participants were asked whether their hospitals used social media to engage consumers in any service design or QI activities. More than half of the participants reported that social media had been used at their hospital as a consumer engagement tool. Most common was the use of social media to recruit people to consumer representative roles or to consultation activities that occurred off social media platforms (e.g., surveys, face-to-face activities). The use of closed or private social media groups as virtual spaces for consultation or co-design with consumers was reported by three participants, and two participants reported that their hospital had asked for public feedback on QI projects through social media platforms. Three participants also reported that consumers had been involved in planning aspects of the hospital’s overall social media strategy.

No participants reported that their hospital formally collected patient experience data via social media to inform service design or QI projects. However, when this was explored further it was clear that some service provider participants were aware of patients, carers and family members occasionally using social media as a channel to give feedback to the hospital. Feedback provided through social media was handled differently by different services, and positive and negative consumer feedback was sometimes handled differently within a service.

Typically, if a person made a complaint or described a negative experience to the hospital via social media, they would be directed off social media and into other hospital feedback mechanisms. Often it was then up to the complainant to make the complaint again through this new mechanism.

If someone’s got a … clinical care issue, or a complaint, then we’ll just private message people back and suggest that they might like to contact our … patient liaison service, and they can help look into things that way. And then it’s up to them if they wish to pursue that. (CO3)

Some participants described a more proactive process for dealing with complaints made through social media, either by passing the original complaint on to the patient experience department directly, or by staff offering to help the complainant navigate the complaints process.

Complaints, comments, thoughts, happy feedback, positive feedback it all gets fed back through our customer relations team and then on to the relevant departments. We don’t really engage in that space, but we certainly facilitate the feedback, positive, negative or whatever, gets back to the right area and is responded to. (CO4)

In contrast, participants reported that positive feedback shared through social media was sometimes passed directly to the area of the hospital or staff member being complimented.

A patient messaged just to say he’d had fantastic care from some of the … staff on one of the wards … and he wanted me to pass on the gratitude. … So I reached out to the nurse unit manager and … forwarded this patient’s comments, and the nurse unit manager just responded straight away and said ‘that’s great, it’s so nice to receive positive affirmation’. (CO1)

Very few participants were able to describe a mechanism for how feedback received through social media was used to inform service design and QI. However, most participants believed that the data was collated and analysed by QI departments.

I don’t work in the quality section, but … I would say that … because it goes through the … consumer liaison officer who does complaints and compliments, and they’re part of the quality and safety team, … that would go into their data. (CE4)

One participant was very frank about their hospital’s handling of patient feedback received through social media.

We don’t have a standard process of collecting that information at all, and nor are we out there seeking feedback on particular issues. (QI1)

### The benefits and risks of social media as a tool for consumer engagement in hospital service design or QI activities

The benefits and risks discussed by interview participants were grouped into five themes. The benefit of *overcoming barriers to engagement* was identified by consumer and service provider users of social media. The potential for social media to facilitate *consumer-initiated engagement* was viewed by participants as having particular benefit for consumer users. The themes of *breadth* vs *depth of engagement* and *organisational transparency* vs *control* describe both benefits and risks of social media use identified by participants. These themes highlight the conflicting views around the quality of information and engagement possible through social media, and the impact of social media-based engagement on organisational reputation. The final theme, *users causing harm,* explores the risks of actual harm caused by the actions of individual social media users.

#### Overcoming barriers to engagement

Most participants believed that the use of social media for consumer engagement reduced or overcame barriers associated with more typical face-to-face engagement methods.

For consumers, social media engagement could be used in place of physical attendance at face-to-face engagement activities. Consumers who could benefit from social media engagement methods included people whose illness or disability made leaving the home difficult, working people, people living far away from the hospital, people with young children and other carer responsibilities, and people who have issues accessing transport.

Not everyone can physically get into the hospital to give their opinion or attend a focus group, so I think it’s really important that a hospital does do a lot of different avenues of consulting with the community, and social media would be one way to do that, particularly to capture the opinions of working people, or even just of people who are too sick to come out of home, but have got quite legitimate and relevant opinions about how their services are being received by them, and how they would like it to be improved. (CE2)

Social media was considered low cost, or cost effective, compared to other methods of engagement, and could therefore overcome financial barriers for consumers with limited finances, and consumer engagement projects with limited budgets.

I’m thinking of those people who are … thinking about budget, and thinking ‘what’s going to be the best bang for buck, the quickest possible way to achieve the getting of information’. (CE1)

If you’ve got the set up and you know how to use it, it is cheap, easy, accessible. (CR7)

Many participants talked about social media being widely used and well understood, or easy to learn for new users. It was seen as an efficient way to run consumer engagement activities because time and effort didn’t need to be spent supporting consumers or providers to access or understand the technology.

Everyone’s on social media these days, it seems to be the community hub, social media, doesn’t it? It’s just what we do these days, it just makes perfect sense to me. (CR4)

#### Consumer-initiated engagement

Participants identified that social media allows consumers to provide quality of care information more easily and directly, which could inform service design and QI activities. Some participants believed that social media allowed consumers to speak directly to the service, rather than communications being channelled and reinterpreted through other feedback or consultation mechanisms.

I see some potential … about bringing in the consumer voice so that when hospitals … want a consumer community voice, … it’s a very accessible medium and an easy medium for many people to be able to have some input. (CR7)

Social media was also seen by some participants as an avenue for consumers to provide feedback or communicate with the hospital when other feedback or communication mechanisms fail.

Consumer representative participants identified benefits of social media in relation to their role. It was seen as an easy point of first contact, or way to build relationships, for people seeking a consumer representative role within a hospital.

So you can maintain that relationship online or digitally, and then hopefully when you get a chance to come to … the place you’re engaging with, then it’s not like ‘who are you?’ It’s like ‘that’s Mike, or Suzy, or Mohammed who kept in touch with us. Oh great to meet you!’. So you kind of have that ‘in’ in a way. (CR3)

Some consumer representatives had also used social media to connect with other consumer representatives. Social media was used between consumer representatives for support, knowledge sharing, and collectivisation and advocacy around specific issues.

Social media also gives an opportunity for patients, carers and community to actually engage independently of the health service, to give … an individual or a collective voice back. (CR7)

#### Breadth vs depth of engagement

Most participants spoke about the benefit of increased breadth of consumer engagement. Social media could engage more people in service design and QI activities and had the potential to reach a greater diversity of people than typically involved in consumer engagement. Participants believed that social media could be used to engage more with young people, non- or intermittent- service users, culturally and linguistically diverse communities, people with disabilities and chronic illness, and Indigenous people.

Diverse patients, those of different cultural backgrounds, may not like the traditional face-to-face sort of thing. I think social media is underutilised by health services in gaining that feedback, and there are groups who are marginalised because we don’t. (CE5)

Participants frequently spoke about the potential to use social media to engage with the community local to the hospital.

I think the more that we can engage with the community on social media is a benefit for us. … We want to increase our engagement with local people, with people across [city], with people across the world if they want to, but especially our local community, we want them to engage with the health service … and then subsequently their input and opinions and thoughts. (CO4)

Many participants emphasised the speed of engagement possible through social media. Gathering a large range of opinions more quickly than through typical face-to-face engagement was considered beneficial.

… to reach a larger voice, reach a larger sort of community. … social media provides a great platform to do that. Whereas … going through traditional means would be time consuming. (CO1)

Social media engagement being fast and far-reaching was seen by a few participants to be at odds with the relationship building and rich information they believed was required for high quality consumer engagement in service design and QI. These participants believed that information shared on social media was often superficial and not suitable for QI purposes, social media platforms did not support co-design models of engagement, and social media was often overrun by low value ‘noise’.

To have that … facilitated discussion in a room, with people where you can hear their experiences and talk through how it works from both perspectives is really valuable for the participants and the staff who are involved in it. But on social media … you can’t have that sort of depth of discussion. (CO3)

Some participants also raised concerns that despite the breadth of reach, social media audiences might not meet the hospital’s consumer engagement needs. These participants identified that social media-based engagement activities exclude people who don’t use social media, and the broad reach could mean people who engaged with hospital social media accounts may not actually represent the service population of the hospital.

One risk is that we might not actually be targeting the right consumers, or people who live elsewhere in state or country do still have the ability to use those channels to influence the work we do. (CR12)

#### Organisational transparency vs control

The use of social media for consumer engagement in QI and service design was seen to bring both risks and benefits for the hospital’s reputation. Social media-based consumer engagement could increase organisational transparency, but also required hospitals to surrender some control of feedback mechanisms. Tensions between organisational transparency and control were generally discussed in the context of public social media channels, rather than private groups.

Increased organisational transparency through social media could increase consumer trust and help build a hospital’s reputation.

One of the first things when the administrator came in was that … the board had lost touch with the community and we needed to build our connections with the community. And they need to be able to see what’s going on to rebuild trust. So part of that was social media. (CO3)

Social media could also help improve hospital reputation through broadcasting positive stories about hospital activities.

It’s very much two ways. Not only are they able to communicate with us through our platforms, but also we’re able to enhance our reputation through our communication, through messages about the hospital. (CO2)

Additionally, simply having social media accounts was seen by some participants as an indicator that a hospital was up-to-date, modern and approachable.

If the organisation says ‘well we don’t do that stuff, we’re too serious, this is a serious business, healthcare’ then the perception we’re not in touch with people or that we don’t actually care about modern ways of doing things I think is a real risk in terms of who might feel willing to put their hand up and say ‘I want to let you know how you could do something better’. (QI3)

However, most participants also identified reputational risks associated with a potential loss of control over patient feedback and consumer engagement mechanisms, particularly if negative patient stories were shared on public social media pages.

If there were multiple people complaining it could really skew the community’s perception of the health service. When … there might be hundreds of thousands of consumers that have had quite fine experiences and are quite neutral either way. (CR10)

Some participants also believed that complaints received through social media didn’t provide useful information for QI and service design. People perceived as frequent complainers, or people who “vented” or “ranted” on social media, were considered non-genuine in their complaints, and of limited use for informing service changes.

The ones who want to just continually complain, even though their issues may have been addressed, they still have a negative view. They’re the half glass empty people who just get on and complain and complain and complain and won’t necessarily provide us with any information that is useful to influence what we’re doing. (CE3)

In addition, two participants believed that the possibility of consumer representatives collectivising through social media and making demands on the hospital was a risk for their organisation.

Despite these concerns about complaints given in public social media spaces, there was recognition by some participants that there may be advantages for consumers providing feedback in a public way through social media, rather than through the private communication common to other hospital feedback mechanisms.

If I were a consumer I’d want other people to know that this has gone wrong. And so if there were public comments rather than private comments then others might be able to comment and support or change the conversation slightly, and more effectively inform both parties. (QI4)

#### Users causing harm

Almost all participants talked about the risk of social media users acting maliciously and causing harm to other users, particularly if social media spaces were not adequately moderated. These harms could come from consumer users, service provider users, and users not associated with the hospital. Malicious actions included bullying, harassment, aggression and trolling.

Bullying, harassment, all of that sort of stuff, can potentially come out … or it can be used as a medium for those sort of things. (CE3)

Participants also identified other malicious actions such as hacking, behaving poorly because of the ability to be anonymous, and users misrepresenting themselves.

Some participants raised concerns that a user may harm themselves or others in response to a post or interaction on social media.

If someone reads something and they don’t read it clearly and it causes them distress and they take action on their distress, that could be a very negative outcome. (CE1)

Breaches of staff and patient privacy were identified as a risk which could cause direct harm to individuals or organisations. Health providers had concerns that their private life could be revealed to patients or colleagues, or that people could easily share a patient’s information without consent. Consumers raised the risk of being identified as a service user when interacting with services on social media.

If you’re applying for a job and everything goes very smoothly in the last stage, the HR access [es] your social media and finds you have a history of mental health disorder, they might just say ‘why should I recruit you, you could cause trouble in the future’. (CR1)

Related to breaches in privacy, breaches in professional behaviour on social media were identified by some participants as being a potential source of harm for the organisation. Service providers sharing confidential information about colleagues or patients, or behaving inappropriately on social media while being identifiable as hospital employees, were potential breaches of professional behaviour.

We do have a lot of staff who follow us on social media, if they have listed where they work, and then they engage in behaviour on other social media pages which isn’t considered appropriate, we’ve had complaints about staff in that respect via social media, from various consumers. (CO1)

Finally, legal risks, such as defamation or users discussing illegal activities on hospital social media pages, were recognised by some participants as having the potential to cause direct harm to an organisation.

## Discussion

The key finding from this research is that social media is being used for some consumer engagement functions in hospital service design and QI activities. Social media was used to recruit consumers to engagement activities (including committees, surveys), for seeking feedback on QI projects, and as virtual private ‘rooms’ for consultation or co-design activities. Consumers were also using social media to provide feedback on healthcare experiences which could inform future activities, and create new avenues or mechanisms for obtaining valuable insights from service users that improve healthcare quality and safety [18]. Social media could help overcome the barriers of more typical face-to-face methods of engagement, but could also lead to harms through negative or malicious actions by users. These risks and benefits identified in this study align with previously identified risks and benefits of health-related use of social media from Australian and international studies [30–35] and risks of use identified in the broader social media research literature [36]. However, two of the themes – breadth vs depth of engagement, and organisational transparency vs control - reveal areas of tension within the participants’ experiences or perceptions of benefits and risks that add new perspectives to the research.

While the reach of social media may increase the number and diversity (‘breadth’) of consumers who can engage in hospital service design and QI activities, some participants expressed concerns that the quality of information and relationships built (‘depth’) might be compromised. Participants perceived that ongoing engagement over time and in-depth discussions with consumers was not possible through social media. There was also concern that social media-based initiatives would exclude people who were not social media users or would fail to engage the ‘right consumers’.

While it is important that the potential risks around the quality or effectiveness of social media engagement are identified, traditional face-to-face consumer engagement activities also incur similar risks if not designed and delivered effectively. Previous research into face-to-face methods of engagement identifies similar risks of tokenism or symbolic participation [37–39]. Building partnership and collaborations between a diverse range of providers and consumers are often viewed as markers of high-quality consumer engagement in health service design and QI [39–41]. However, consumer engagement in health commonly occurs in models where the organisation establishes the space for engagement and controls the topics discussed, the ways consumers engage, and ultimately the decisions that are made [42, 43]. This can lead to tokenistic forms engagement where consumers have no real power to influence change or make decisions [37, 38], in contrast to genuine partnerships between consumers and providers. As a result, forms of engagement where patients are consulted rather than being involved as decision-making partners, such as surveys or focus groups, are often more typical than partnership approaches, such as co-design [39, 44]. Similarly, a lack of diversity is common in consumer engagement activities no matter the method of engagement, with hospitals often only, or more commonly, involving people in consumer engagement activities who are white, middle class, and have good health literacy [39, 45]. Social media is sometimes discussed in the literature as a potential way to overcome a lack of diversity in consumer engagement activities [45–48], but its current use in service design and QI is likely not meeting that theoretical potential [6].

The tension for organisations between transparency and control was a key finding. Social media was often perceived by participants as not the right avenue for complaints. Receiving complaints in a public forum was seen as a reputational risk for the organisation, and social media-based complaints were often viewed as not valuable for informing service design and QI activities. As a result, hospitals in this study often responded to feedback given through social media by trying to re-direct complainants off social media, to less public, feedback pathways. This conflicted with the study participants’ beliefs that social media was a good platform for consultation and feedback, for accessing an authentic consumer voice, and for improving a hospital’s reputation. It was also acknowledged that consumers might be more confident or willing to provide feedback if they can do so using social media platforms.

These conflicting views and experiences may reflect a broader issue– that many hospitals and health services have not yet established good systems for responding to patient feedback, and integrating it into service design and QI activities, regardless of the mechanism through which it’s received [49–52]. The lack of clarity around the link between patient feedback and QI was seen in these interviews, with many participants assuming that patient experience feedback received through social media would be used to inform service design or QI, but most being unable to describe exactly how that happens. Previous studies have found that negative provider or organisational attitudes to patient feedback, including scepticism about the reliability of patient complaints as a source of data [49, 53], fears that feedback will be used punitively [49] and concerns about organisational reputation [52], can act as barriers to using feedback data to inform QI. These same attitudes are reflected in the risks identified by many participants of this study. Negative perceptions of patient complaints may be heightened by the potentially public nature of feedback given through social media. However, the fact that previous studies which did not include social media found similar attitudes and problems with using consumer feedback to inform QI, indicates that, rather than being specific to this study or social media-based consumer engagement, this issue is likely a broader tension that exists around how (or if) consumers can influence health services.

### Opportunities for future research and implications for practice

This article presents the findings from an interview study exploring the use of social media as a tool for consumer engagement in Australian hospital service design and QI activities focusing on methods, risks, and benefits of use. Given that this was the first Australian study of its kind to our knowledge, with a small cohort of participants, and conducted in the dynamic context of social media, there are opportunities for future research with larger numbers of participants and other health settings, to ensure we keep learning about the experiences, outcomes and impacts of social media use in health.

Additionally, while the scoping review demonstrated a lack of Australian studies around social media-based engagement [6], it also revealed that only a few of the included studies conducted an in-depth examination of the experience of people using social media for engagement [9, 10, 14, 16, 54]. This presents an opportunity for other researchers to partner with health services who are implementing social media-based engagement and apply the methods outlined in this study to better understand the experiences of consumers and service providers involved in social media-based consumer engagement in their specific contexts.

In terms of implications for practice, this study presents a range of risks and benefits which should be considered by people who are planning and implementing social media-based consumer engagement in service design and QI activities. Given that the findings align with many of the known risks and benefits of face-to-face consumer engagement and/or health-related social media use from the international literature, it is likely that the findings of this study are applicable to health systems outside of Australia, particularly public health systems in high income countries.

Additionally, this study identified that some hospitals and health services have already implemented social media-based consumer engagement activities. Despite the risks discussed, participants were largely in favour of hospitals integrating social media-based engagement into service design and QI activities. Therefore, hospitals which are already undertaking social media-based consumer engagement activities should consider sharing their methods and knowledge with other hospitals and health services. Case studies from health services outlining how social media has been integrated into consumer engagement in service design and QI could assist organisations interested in trying these methods to translate this research into practice.

Finally, the findings of this study add to the evidence base about long-standing issues in consumer engagement around how hospitals can more effectively partner with a diverse range of consumers and use feedback to inform service design and QI activities. If hospitals use social media methods of engagement it is important that they understand that social media use alone is not a panacea for a lack of diversity or quality consumer engagement. Those responsible for consumer engagement and use of patient feedback in hospitals instead need to look critically and holistically at how consumers and their data are integrated into service design and QI. Systems and processes which support deep, meaningful and representative consumer engagement are needed, regardless of the engagement method used.

### Limitations

The study inclusion criteria required participants to have interest in using social media for consumer engagement in health service design and QI, but they were not required to have direct experience. As a result participants had varied levels of experience with using social media for consumer engagement in service design and QI activities, and some participants had no experience. During data analysis, the frequency of codes, categories and themes were compared between those who had experience and those who did not to check for differences in perspectives and there were no major differences. This indicates that even when people do not have experience in using social media for consumer engagement in health service design or QI projects, they are able to anticipate potential risks and benefits similarly to those who do already use social media.

Secondly, this study did not capture the views and experiences of participants who were not social media users and/or not interested in using social media as part of engaging consumers in hospital service design and QI. Including these participants could have provided additional insights, particularly around the perceived risks of social media, however this was beyond the scope of the study but could be explored in further research. Finally, it should be noted that interview participants were not involved in member checking transcripts or how data were coded.

## Conclusion

This study demonstrated that the use of social media as a tool for consumer engagement in Australian public hospital service design and QI activities is viewed as having the potential to benefit a range of stakeholders, particularly if the identified risks can be mitigated. Areas of tension were also revealed, the two most significant being the risk that social media-based consumer engagement sacrifices depth for breadth, and that organisations find it difficult to balance the benefits and risks of maintaining control over communications and increasing transparency. However, these tensions are not unique to social media based-consumer engagement. Problems such as tokenistic styles of engagement and organisations struggling to balance control of information with how consumers influence service design and QI, are common even when social media is not being used as a method of engagement. Therefore, it is important for people who are involved in consumer engagement activities to understand that social media-based forms of engagement are unlikely to either create, or solve, issues with consumer engagement that hospitals face. Instead, those issues need to be taken into consideration, and strategies should be designed to manage them, regardless of the methods used for engagement.

## Data Availability

The datasets generated and/or analysed during the current study are not publicly available due to privacy concerns, but are available from the corresponding author on reasonable request.
